# Phytochemical Components and Bioactivity Assessment among Twelve Strawberry (*Arbutus unedo* L.) Genotypes Growing in Morocco Using Chemometrics

**DOI:** 10.3390/foods9101345

**Published:** 2020-09-23

**Authors:** Hafida Zitouni, Lahcen Hssaini, Zerhoune Messaoudi, Hamza Ourradi, Manuel Viuda-Martos, Francisca Hernández, Sezai Ercisli, Hafida Hanine

**Affiliations:** 1 Laboratory of Bioprocess and Bio-Interfaces, Faculty of Science and Technics, University Sultan Moulay Slimane, BO 523 Beni-Mellal, Morocco; hafiida2012ziitn@gmail.com (H.Z.); ouradihamza@gmail.com (H.O.); 2 Research Unit of Plant Breeding and Plant Genetic Resources Conservation, National Institute for Agricultural Research (INRA), BO 578 Meknes, Morocco; hssaiini@gmail.com; 3 Departement of Arboriculture, Horticulture and Viticulture, National School of Agriculture, (ENA), BO S/40 Meknes, Morocco; messaoudiz@yahoo.fr; 4 Dpto. Tecnología Agroalimentaria, IPOA. Escuela Politécnica Superior de Orihuela (Universidad Miguel Hernández), Ctra Beniel, km 3.2, E-03312 Orihuela (Alicante), Spain; mviuda@umh.es; 5 Dpto. Producción Vegetal y Microbiología, Grupo de Investigación de Producción Vegetal y Tecnología, cuela Politécnica Superior de Orihuela (Universidad Miguel Hernández de Elche), Ctra. de Beniel, km 3.2, E- 03312 Orihuela, Alicante, Spain; francisca.hernandez@umh.es; 6 Department of Horticulture, Agricultural Faculty, Ataturk University, 25240 Erzurum, Turkey; sercisli@gmail.com

**Keywords:** *Arbutus unedo* L., lolyphenolic compounds, antioxidant capacity, chemometrics, Morocco

## Abstract

There are not many exhaustive works emphasizing the amount of genetic diversity among the strawberry tree (*Arbutus unedo* L.) genotypes in Morocco. This work aims to assess the biochemical composition of strawberry tree fruits, as well as to establish the variation of this composition among them. In this study, total phenols (TP), total flavonoids (TF), condensed tannins (CT) and hydrolyzable tannins (HT), total anthocyanins (TA), and free radical scavenging activity through ABTS were investigated in strawberry tree fruits. Furthermore, qualitative and quantitative analyses of individual phenolic compounds by high-performance liquid chromatography (HPLC) were carried out. Color parameters such as lightness (L*), Chroma (c*), and hue angle (h°) were also investigated. All studied variables showed highly significant differences among all samples with the exception of hydrolyzable tannins and chromatic coordinates. TP varied from 22.63 ± 1.74 to 39.06 ± 2.44 mg GAE/g DW, TF varied from 3.30 ± 0.60 to 8.62 ± 1.10 mg RE/g DW, and TA ranged between 0.12 ± 0.06 and 0.66 ± 0.15 mg cya-3-glu/100 g DW. In addition, CT and HT amounts were in the range of 10.41 ± 1.07–16.08 ± 1.50 mg TAE/g DW and 4.08 ± 2.43–6.34 ± 3.47 TAE/g DW, respectively. Moreover, the IC50 value (ABTS) ranged between 1.75 and 19.58 mg AAE/g DW. 17 phenolic compounds were detected in strawberry tree fruits. Gallocatechol and catechin were the most abundant phenolic compounds. Matrix of correlations revealed significant positive and negative correlations among variables particularly c*, a*, and b*. Principal component analysis (PCA) showed that the first three components formed than 68% of the total inertia. The following variables gallic acid, protocatechuic, gallocatechin, gallic acid derivative, chlorogenic acid, syringic acid, ellagic acid derivative II, L*, and h* were the most involved in the total variance explained. Hierarchical clustering classified samples into one main cluster, with a single branch. The results highlight a high biochemical diversity within studied strawberry genotypes, which is probably more genetically related.

## 1. Introduction

The strawberry tree (*Arbutus unedo* L.) is a wild fruit tree belonging to the Ericaceae family and the genus Arbutus. It is an evergreen fruit tree distributed in the Atlantic-Mediterranean region mainly in southern Europe, North Africa, Ireland, Palestine, and Macaronesia [1]. This plant can grow at different altitudes, from sea level to 1200 m, in various types of soils, but preferably acidic soils [2].

Strawberry tree is frequently used in traditional medicine in some countries such as Spain and Morocco [3,4]. It is known for its diuretic, antiseptic, and laxative effects as well as for its uses in the treatment of cardiovascular pathologies such as hypertension, atherosclerosis, and thrombosis [5,6,7]. The potential health-promoting properties are mainly related to the antioxidant capacity provided by phenolic compounds such as flavonoids, tannins, vitamins (C and E), and carotenoids [8,9,10,11,12,13]. Fruits of strawberry tree contain different phenolic compounds, namely gallic acid [14,15], protocatechuique acid, gentitic acid, phydroxybenzoic acid, vanillic acid, m-anisic acid, arbutin, ß-D-glucogallin, gallic acid 4-O-ß-D-glucopyranoside, 3-Ogalloylquinic acid, 5-Ogalloylquinic acid, 3-O-galloylshikimic acid, and 5-O-galloylshikimic acid.

In the past, a few studies were conducted to demonstrate the genetic diversity among strawberry tree genotypes from Turkey, Spain, and a few other countries [16,17,18]. Morphological and biochemical markers have been widely used in fruit trees valorization and in the investigations into diversity of species and the relationship between genotypes, cultivars, and their wild parents. More recently, biochemical content, in particular, bioactive content of fruits has been widely searched in terms of their human health benefits. The growers are now searching to find genotypes that have higher bioactive content in order to use them to select new cultivars that possess high nutrient value for Human health [19].

In Morocco, strawberry tree fruits remain underexploited and their consumption lasts seasonal. To our knowledge, there are no scientific studies yet studying biochemical variability among strawberry tree genotypes under Moroccan ecological conditions. Moreover, phenolic compounds and fruit skin color measurements were rarely included in previous works on strawberry tree characterization. In the present work, twelve strawberry tree genotypes, belonging to several areas in Morocco, were characterized according to their biochemical markers and skin color coordinates.

The main objectives of this study were: (1) to assess the biochemical composition and colorimetric characteristics of strawberry tree fruits; (2) to determine the correlations between all parameters in order to provide information about the ones that are potentially important in assessing strawberry tree genotypes; and (3) to evaluate the biochemical diversity among the strawberry tree genotypes belonging to several areas in Morocco. The genetic variability determined in this study will facilitate strawberry tree breeding and identification of genetic determinants of trait variability.

## 2. Materials and Methods

### 2.1. Plant Material

Fruits of strawberry tree (*Arbutus unedo* L.) were harvested during the period between October and November of 2019 from several regions of Morocco where they grow naturally (Table 1). At each site, three random samples of fruits were harvested at their full maturity from 30 randomly selected trees. Random samples of fruits were established with approximately 500 g fruits each.

All selected berries had no diseases and visual blemishes. The samples were frozen at −20 °C, freeze-dried, and ground prior to the analyses.

### 2.2. Chemicals and Reagents

Gallic acid, rutin, Folin Ciocalteu reagent, ascorbic acid, and sodium carbonate (Na₂CO₃) were purchased from Sigma-Aldrich (St. Petersburg), ABTS [2,2′-azinobis-(3-ethylbenzothiazoline-6-sulfonic acid)] was from HIMEDIA, tannic acid, and potassium iodate was from Scharlau. Standard compounds (phenolic acid standards: gallic acid; protocatechuic; gallocatechin; catechin; cholorgenic acid; syringic acid; ellagic acid; cyanidin-3-glucoside; quercetin-3-xyloside; rutin; quercetin-3-galactoside; quercetin-3-glucoside; cyanidin-3,5-diglucoside; cyanidin-3-arabinoside; and gallic acid) were obtained from Sigma-Aldrich (St. Petersburg) and from Extrasynthese (Genay, France), and the water was distilled and filtered through a Milli-Qapparatus filter.

### 2.3. Extraction Procedure

One gram of powder from each sample was mixed with 25 mL of ethanol (1:25, *w/v*) at 25 °C for 15 min using an IKA T-18 digital Ultra-Turrax homogenizer. The homogenate was then centrifuged for 10 min at 6000 rpm and the supernatant was removed from the residue. The latter was homogenized with ethanol and the supernatant removed as above. The supernatants were then combined and filtered.

### 2.4. Total Phenols (TP)

TP was determined by using the Folin Ciocalteu method [20]. Briefly, 100 µL of diluted sample (1/100) with ethanol was added to 400 µL of 1/10 diluted Folin Ciocalteu reagent. After 5 min, 500 µL of 10% (*w/v*) sodium carbonate solution was added. After 1 h of incubation at room temperature, absorbance at 765 nm was measured in triplicate with a spectrophotometer (UV/visible, Spectraphysic Model JASCO series V—630 instrument, JASCO corporation 2967-5 Ishikawa-matchi Hachioji-shi, Tokyo 192-8537, Japan). The TP was expressed as gallic acid equivalent per dry weight of strawberry tree fruit (mg GAE/g DW).

### 2.5. Total Flavonoids (TF)

TF was measured using the colorimetric method with aluminum chloride [21]. First, 1 mL of the sample was diluted separately then mixed with 1 mL of a 2% aluminum chloride solution. The mixture was incubated at room temperature for 15 min. Rutin was used to develop the calibration curve. The absorbance was measured at 430 nm with a spectrophotometer (UV/visible, Spectraphysic Model JASCO series V—630 instrument, Japan). The results were expressed as rutin equivalent per dry weight of strawberry tree fruit (mg RE/g DW).

### 2.6. Condensed Tannins (CT)

The CT were determined according to the colorimetric method of Folin Denis [22]. Briefly, 75 mL of distilled water, 1 mL of diluted extract, 5 mL of Folin Denis reagent, and 10 mL of saturated solution (Na₂CO₃) were introduced into 100 mL vial. The saturated solution (Na₂CO₃) was prepared from 43.75 g of sodium carbonate dissolved in 100 mL of hot water (70 to 80 °C) and after cooling, the solution was filtered and adjusted to 125 mL. After mechanical stirring, the preparation was left to stand for 30 min and the optical density was measured at 760 nm with a spectrophotometer (UV/visible, Spectraphysic Model JASCO series V—630 instrument, Tokyo, Japan). A tannic acid standard range was prepared under the same conditions. The results were expressed as tannic acid equivalent per dry weight of strawberry tree fruit (mg TAE/g DW).

### 2.7. Hydrolyzable Tannins (HT)

HT were determined according to the method described by Willis and Allen [23]. Briefly, 5 mL of KIO₃ solution (2.5%) were placed in test tubes, which were then placed in a water bath at 25 °C. 1 mL of diluted extract or standard was added and stirred for 10 s, then the tubes were returned to the water bath. After the optimum time (4 min) had elapsed, the absorbance was measured at 550 nm using a spectrophotometer (UV/visible, Spectraphysic Model JASCO series V—630 instrument, Tokyo, Japan). A tannic acid standard range was prepared under the same conditions. The results were expressed as tannic acid equivalent per dry weight of strawberry tree fruit (mg TAE/g DW).

### 2.8. Total Anthocyanins (TA)

TA content was quantified according to the pH differential method using two buffer systems: Potassium chloride buffer pH 1.0 (25 mM) and sodium acetate buffer pH 4.5 (0.4 M) [24,25]. Briefly, 1 mL of the extract was mixed separately with 4 mL of each of the two buffers. The absorbance was measured at 510 and 700 nm with a spectrophotometer (UV/visible, Spectraphysic Model JASCO series V—630 instrument, Tokyo, Japan) after 15 min of incubation at room temperature. The TA of samples (mg cyanidin-3-glucoside equivalent/100 g DW) was calculated by the following Equation (1):

TA = (A * MW * DF * 1000/Ɛ * L)(1)

where, A: Absorbance = [(A_510nm_ − A_700nm_)]_pH_1.0__ − [(A_510nm_ − A_700nm_)]_pH_4.5__; MW: Molecular weight (449.2 g/mol); DF: Dilution factor; Ɛ: Molar absorptivity coefficient of cyanidin-3-glucoside (26,900 L/mol cm).

### 2.9. Determination of Antioxidant Capacity

The antioxidant activity was evaluated using ABTS [2,2′-azinobis-(3-ethylbenzothiazoline-6-sulfonic acid) assay and the results were presented as mean ± standard deviation. The method used was described by Dorman and Hiltunen. [26]. The ABTS cation radical was prepared by mixing an equal volume of potassium persulfate solution (2.45 mM) with stock solution of ABTS (7 mM). After 16 h of incubation, the solution was diluted with ethanol to give 0.7 to 0.8 absorbance at 734 nm. Then, 10 µL of this freshly prepared solution were added to 990 µL of extract and absorbance was measured at 734 nm with a spectrophotometer (UV/visible, Spectraphysic Model JASCO series V—630 instrument, Tokyo, Japan). Following this, 10 µL of this freshly prepared solution were added to 990 µL of extract and absorbance was measured at 734 nm after 6 min of incubation. The results were expressed as mg ascorbic acid equivalent per dry weight of strawberry tree fruit (mg AAE/g DW).

### 2.10. Extraction and Determination of Polyphenolic Compounds

#### 2.10.1. Extraction Method

Samples (1 g) were mixed with 10 mL of methanol: Water (80:20, *v/v*) and then the mixtures were sonicated during 30 min and macerated one h in refrigeration (4 °C). After this time, the samples were centrifuged for 10 min, 8000 g at 4 °C. The supernatants were collected, and the pellets were mixed with 10 mL of acetone:water (70:30, *v/v*) and the same steps were repeated (sonication, maceration, and centrifugation). Then, the supernatants were combined and evaporated to dryness using a rotary evaporator R-205 (Büchi, Flawil, Switzerland) under reduced pressure, at 40 °C. Then, 5 mL of methanol were added to the residue, and the mixture was well shaken in a stirrer for 2 min. Due to the high sugar content present in the samples, which could interfere with the HPLC column, the samples were loaded onto a C18 Sep-Pak cartridge, previously conditioned with 5 mL of methanol, 5 mL of pure water, and then with 5 mL of 0.01 mol/L HCl. The cartridge was washed with 5 mL of pure water and then eluted with acidified methanol (0.1 g/L HCl). The collected fractions were stored at −20 °C until further use.

#### 2.10.2. Determination of Polyphenolic Compounds

**Figure 1:**
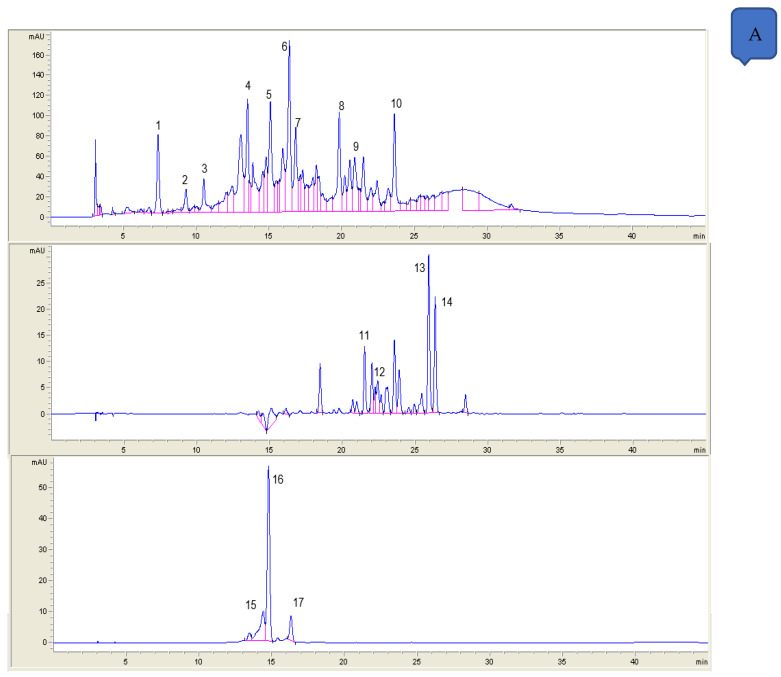

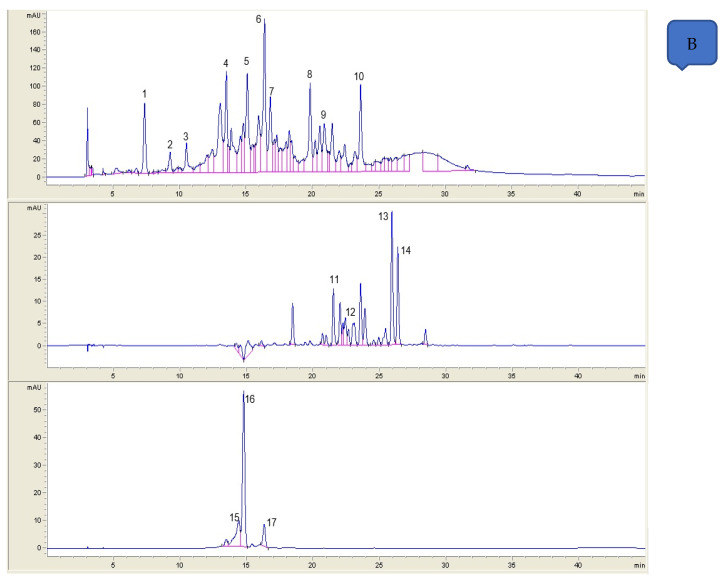
Chromatographic profiles at different acquisition «lambda: 280nm; lambda: 320nm; lambda: 360 nm and lambda: 520 nm»: (**A**) MDZ strawberry genotype and (**B**) TAH strawberry genotype.

Polyphenolic profiles of all samples were determined by high performance liquid chromatography (HPLC) [27]. A volume of 20 µL of the samples were injected into a Hewlett-Packard HPLC series 1200 instrument (Woldbronn, Germany) equipped with a diode array detector (DAD) and a C18 column (Mediterranea sea 18, 25 × 0.4 cm, 5 micrometers particle size) from Teknokroma, (Barcelona, Spain). Polyphenolic compounds were analyzed in standard and sample solutions using a gradient elution at 1 mL/min. The mobile phases were composed by formic acid in water (1:99, *v/v*) as solvent A and acetonitrile as solvent B. The chromatograms were recorded at 280, 320, 360, and 520 nm (Table 2). Polyphenolic compounds identification was carried out by comparing UV absorption spectra and retention times of each compound with those of pure standards injected in the same conditions (Figure 1). The compounds were quantified through calibration curves of standard compounds injected in the same conditions. Phenolic acid standards were dissolved in methanol at different concentrations between 10 and 200 μg/mL; flavonoids standards were dissolved in methanol at different concentrations between 1 and 250 μg/mL. Quantification of anthocyanins was carried out based on linear curves of authentic standards. A cyanidin 3-glucoside calibration (concentration between 1 and 250 μg/mL) was used for cyanidin derivatives.

### 2.11. Skin Coordinates Color

Color determinations were made on fresh strawberry tree genotypes, at 25 ± 1 °C, using a NH310 colorimeter (3 nh, Model YS3010, Shenzhen 3NH Technology, Co., Ltd., Shenzhen, China). This spectrophotometer uses a Xenon lamp, illuminant D65, 10° observer, SCI mode, 11 mm aperture of the instrument for illumination and 8 mm for measurement. Color data were provided as International Commission on Illumination (CIE) L*a*b* coordinates, which define the color in a three-dimensional space. L* indicates lightness, taking values within the range of 0−100, and a* and b* were the chromatic coordinates, green−red and blue−yellow coordinates, respectively. Parameter a* takes positives values for reddish colors and negative values for the greenish colors, whereas b* takes positive values for yellowish colors and negative values for bluish colors. Color analyses were run in 25 replicates for each block, which means 10 strawberry fruit per treatment. Each measure was examined with three replications.

### 2.12. Statistical Analysis

Since we used different measure, data were standardized (µ = 0 and a σ = 1) so they can have a comparable scale [26]. Prior to the statistical analyses, data were tested for normality and homogeneity of variance using SPSS software v22. The means were evaluated according to descriptive statistics represented as Mean ± SE. Data analysis was performed using IBM SPSS v22. Analysis of variance (One-way ANOVA) was performed to test significant differences among the samples. The differences among means were estimated with Duncan new multiple range (DMRT) test. Correlation coefficients and their levels of significance were calculated using Pearson correlation. Principal component analysis was carried out using correlation matrix. In addition, a scatter plot was created according to the first three principal components (PC1–PC3). A distance matrix generated from biochemical data was used for cluster analysis based on Euclidian distance to better understand the patterns of variability among the samples.

## 3. Results and Discussion

All studied variables showed highly significant differences among all samples (*p* < 0.05), with the exception of hydrolyzable tannins and chromatic coordinates.

### 3.1. Total Phenols (TP)

TP ranged from 22.63 to 39.06 mg GAE/g DW, with an average of 30.20 mg/g DW (Table 3). The highest value was recorded in “LAN” (39.06 mg/g DW) while the lowest value was observed in “OUA” (22.63 mg/g DW). The TP content of strawberry tree fruits reported in this study is higher than those found by other authors; Doukani and Tabak. [28] reported a range of 14.74 to 7.025 mg GAE/g in Algerian strawberry tree cultivars. In another study, Seker and Toplu. [29] reported a TP content ranging from 17.7 to 25.8 mg GAE/g). Also, Colak. [30] and Ruiz-Rodríguez et al. [13] recorded TP values ranging from 483 and 627 mg GAE/100 g and from 951 to 1973 mg/100 g in Turkish and Spanish genotypes, respectively, while Vidrih et al. [19] reported an average of 590 mg/100 g in Croatian fruits.

### 3.2. Total Flavonoids (TF)

TF content ranged from 3.30 to 8.62 mg RE/g DW, with an average of 6.44 mg RE/g DW (Table 3). The highest flavonoids content was observed in “KHN” (8.62 mg/g DW) followed by “TAM” (8.26 mg/g DW) and the lowest value was observed in “KSB” (3.30 mg/g DW). These concentrations are higher than those reported by Jurica et al. [31] (0.23–0.28 mg EQ/g) and Bouzid et al. [32] (2.18–6.54 mg EC/g), and by Pallauf et al. [10] (0.32 mg/100 g edible portion).

### 3.3. Condensed and Hydrolyzable Tannins (CT) and (HT)

CT and HT results data are presented in Table 3. A significant variation of CT was found at (*p* = 0.027) among genotypes. However, there was no statistical difference for HT among genotypes (*p* = 0.998). On the one hand, the CT content ranged from 10.41 to 16.08 mg TAE/g DW, with an overall mean of 13.03 mg TAE/g DW. The highest CT content was observed in “LAN” (16.08 mg TAE/g DW), while the lowest was observed in “BNO” (10.41 mg TAE/g DW). On the other hand, HT ranged from 4.08 to 6.34 mg TAE/g DW, with an overall average of 5.37 mg TAE/g DW. The highest value was found in “CHF” (6.34 mg AT/g DW) while the lowest was recorded in “TAH” (4.08 mg AT/g DW). These values were approximately similar with those revealed by Jurica et al. [31] who found (16.75–18.92 mg GAE/g) for total tannins.

### 3.4. Total Anthocyanins (TA)

The TA content was presented in Table 3. A statistically significant variation at (*p* ˂ 0.01) was observed among the genotypes studied. The TA ranged from 0.12 to 0.66 mg equivalent cyanidin-3-glucoside/100 g DW with an overall mean of 0.34 mg equivalent cyanidin-3-glucoside/100 g DW. The highest TA was observed in “BMR” (0.66 cyanidin-3-glucoside/100 g DW), while the lowest was obtained by “OUA” (0.12 cyanidin-3-glucoside/100 g DW). These values were lower than the ones published by Pallauf et al. [10] (3.77 mg equivalent cyanidine-3-glucoside/100 g).

### 3.5. Antioxidant Activity (AA)

The results obtained for antioxidant activity based on the radical scavenging capacity (ABTS) were reported in Table 3. Significant differences (*p* < 0.001) were observed among the genotypes studied. The value of ABTS assay ranged from 1.75 to 19.58 mg ascorbic acid equivalent/g DW, with an overall mean of 7.49 mg ascorbic acid equivalent/g DW. Gündoğdu et al. [33] analyzed the antioxidant capacity (ABTS) of Turkish strawberry tree fruits. They found values ranged between 17.51 and 30.06 µmol TE/g. In other study, Colak. [30] analyzed the antioxidant capacity (ABTS) of Turkish strawberry tree fruits. They found values ranged between 18.07 and 33.41 μmol TE/g.

### 3.6. Profile of Polyphenolic Compounds

A total of 17 phenolic compounds were identified in strawberry tree fruits. The results obtained were summarized in Table 4. Significant variations in phenolic compounds were found at *p* < 0.001 among genotypes.

Gallocatechol was present in dominant amounts in all genotypes with the exception of “CHF” and “MDZ” where the dominant compound was catechin. The concentration of gallocatechol differed between genotypes. The highest level reported in “OUZ” (79.88 mg/100 g DW) and the lowest in “CHF” (16.15 mg/100 g DW). Catechin was found in higher amounts in all genotypes. “OUZ” had the highest concentration (65.53 mg/100 g DW) of catechin, and “BNO” had the lowest concentration (13.99 mg/100 g DW). Protocatechuic acid was present in significantly higher amounts in “OUZ” (6.98 mg/100 g DW) and significantly lower amounts in “MDZ” (1.84 mg/100 g DW). Gallic acid was present in significantly higher amounts in “OUZ” (58.07 mg/100 g DW), the lowest amount was recorded in “MDZ” (4.56 mg/100 g DW). Gallic acid derivatives were detected in all genotypes. The highest amount was present in “OUZ” (22.02 mg/100 g DW), and the lowest in “CHF” (4.98 mg/100 g DW). The concentration of syringic acid differed significantly between genotypes, with the highest level in “OUZ” (16.55 mg/100 g DW) and the lowest in “CHF” (4.27 mg/100 g DW).

Among the phenolic acid group, chlorogenic acid was significantly higher in all genotypes. The highest level was observed in “TAH” (27.42 mg/100 g DW), and the lowest in “CHF” (5.55 mg/100 g DW). Ellagic acid was also noticed in all genotypes. The highest level was found in “OUL” (39.29 mg/100 g DW) and the lowest in “CHF” (8.42 mg/100 g DW). Ellagic acid derivatives I and II were seen in all genotypes. The highest levels were found in “OUZ” (30.88 mg/100 g DW) and (36.56 mg/100 g DW) respectively, however, the lowest levels were found in “KHN” (7.79 mg/100 g DW) and “CHF” (8.97 mg/100 g DW), respectively. Other minor compounds such as quercetin-3-xyloside, quercetin-3-galactoside, quercetin-3-glucoside, rutin, cyanidine-3-glucoside, cyanidine-3-5-diglucoside, and cyanidine-3-arabinoside were also identified. “OUZ” had the highest amount of quercetin-3-xyloside (7.92 mg/100 g DW), while “MDZ” had the lowest amount (1.43 mg/100 g DW). “KSB” recorded the highest amount of quercetin-3-galactoside (3.46 mg/100 g DW), while “KHN” recorded the lowest amount (1.00 mg/100 g DW). Quercetin-3-glucoside was significantly higher in all genotypes. The highest amount was observed in “TAM” (3.21 mg/100 g DW), and the lowest in “KHN” (0.98 mg/100 g DW). Rutin compound was present in lower amounts in all genotypes. “BMR” had the highest quantity of rutin (2.26 mg/100 g DW) whereas the lowest amount was recorded in “OUA” (0.67 mg/100 g DW). Similarly, cyanidin-3-glucoside was spotted in all genotypes. “TAH” contained the highest amount (7.21 mg/100 g DW) as the lowest was recorded in “OUA” (0.36 mg/100 g DW). Concerning the last two compounds, which are cyanidine-3-5-diglucoside and cyanidine-3-arabinoside, they were identified within only six genotypes. The lowest amounts of them were recorded in “CHF” (0.61 mg/100 g DW and 0.36 mg/100 g DW, respectively) whereas the largest ones were observed in “TAH” (3.30 mg/100 g DW and 1.64 mg/100 g DW, respectively). Our results are consistent with those of Ganhão et al. [34] who had found catechin, gallic acid, ellagic acid, ellagic acid, chlorogenic acid, rutin, and cyanidin-3-glucoside in strawberry tree fruits collected in Spain. However, Ayaz et al. reported that gallic acid (10.7 mg/g DW) was the main phenolic compound in strawberry tree fruits collected in Turkey, followed by protocatechic acid, gentisic acid, *p*-hydroxybenzoic acid, vanillic acid, and m-anisic acid. Distinctively, Mendes et al. [35]. had identified other phenolic compounds in strawberry tree fruits collected in north-eastern Portugal. These compounds are gallic acid glucoside, galloylquinic acid, quinic acid derivative, proanthocyanidin dimer, galloylshikimic acid, digalloylquinic acid, digalloylshikimic acid, catechin monomer, proanthocyanidin trimer, strictinin ellagitannin, ellagitannin derivative, galloyl derivative, trigalloylshikimic acid, myricetin rhamnoside, quercetin glucoside, gallotannin, and ellagic acid rhamnoside.

### 3.7. Skin Color

Color measurements data are reported in Table 5, and there were no statistical differences between strawberry tree genotypes for all color indices L*, a*, b*, c*, and h°. Data showed that Lightening (L*) values ranged from 25.83 to 50.78. a* and b* values ranged from 28.93 to 58.91 and from 70.85 to 93.73, respectively. According to positive values of a* and b*, strawberry tree fruits included reddish orange to deep crimson red fruit colors. The Chroma (c*) was higher in genotypes with clear and bright fruit skin color, where it varied generally between 78.30 and 110.17. The hue angle (h°) ranged between 54.70° and 66.45°. All strawberry tree genotypes were lighter (higher L* values) and tended to be more red (higher a* values) and yellower (higher b* values). Furthermore, the genotypes showed higher values of chroma (c*) and hue angle (h°) corresponding to a lighter color. Therefore, skin color evaluation using these coordinates was of great importance in characterization and assessment of fruits quality and maturity. These results were, globally, in accordance with several studies. Islam and Pehlivan. [36] reported average L*, a*, and b* values of 40 genotypes as 47.26, 37.07, and 26.89, respectively. Also, Colak. [30] reported average L*, a*, and b* values of 15 genotypes as 44.30, 37.53, and 23.88, respectively. According to the literature, the color coordinates was, particularly correlated to the antioxidant compound, essentially phenols (anthocyanins, tannins, catechins, etc.) and carotenoids (lycopene, betacarotene, etc.) [37,38].

### 3.8. Correlation among Variables

In order to identify the relations between biochemical traits, all variables were subjected to bivariate correlation using the Pearson coefficient. Significant correlations at the level of 0.05 or 0.01 are summarized in the Table 6. In the current study, a strong positive correlation was found between condensed tannins and total phenols (r = 0.631; *p* < 0.05). Similarly, links were noticed between protocatechic acid and gallic acid (r = 0.841; *p* < 0.01) as well as between gallocatechin and both gallic acid (r = 0.834; *p* < 0.01) and protocatechic acid (r = 0.913 **). Also, derivatives of gallic acid were correlated to gallic acid (r = 0.717; *p* < 0.01), protocatechic acid (r = 0.854; *p* < 0.01), and gallocatechin (r = 0.841; *p* < 0.01). The correlation between chlorogenic acid and each of the following parameters: Gallic acid, protocatechic acid, gallocatechin, and gallic acid derivatives were significant (*p* < 0.001) and, respectively, 0.651, 0.812 **, 0.806 **, and 0.927 **. The results obtained also showed positive correlations between syringic acid and each of the following parameters: Gallic acid (r = 0.705; *p* < 0.05), protocatechic acid (r = 0.771; *p* < 0.01), gallocatechin (r = 0.764; *p* < 0.01), gallic acid derivatives (r = 0.870; *p* < 0.01), and chlorogenic acid (r = 0.770; *p* < 0.01). In the same way, the study revealed links between derivatives ellagic acid I and gallic acid (r = 0.619 *), protocatechic acid (r = 0.710; *p* < 0.01), gallic acid derivatives (r = 0.821; *p* < 0.01), chlorogenic acid (r = 0.769; *p* < 0.01), and syringic acid (r = 0.590; *p* < 0.05). Correspondingly, it conveyed correlations between derivatives ellagic acid II and gallic acid (r = 0.718; *p* < 0.01), protocatechic acid (r = 0.839; *p* < 0.01), gallocatechin (r = 0.800; *p* < 0.01), gallic acid derivatives (r = 0.976; *p* < 0.01), chlorogenic acid (r = 0.883; *p* < 0.01), syringic acid (r = 0.849; *p* < 0.01), and ellagic acid I derivatives (r = 0.872; *p* < 0.01). As far as ellagic acid concerned, the study portrayed a relationship between it and protocatechic acid (r = 0.757; *p* < 0.01), gallocatechin (r = 0.692; *p* < 0.05), gallic acid derivatives (r = 0.849; *p* < 0.01), chlorogenic acid (r = 0.906; *p* < 0.01), syringic acid (r = 0.590; *p* < 0.05), ellagic acid derivatives I (r = 0.822; *p* < 0.01), and ellagic acid derivatives II (r = 0.847; *p* < 0.01). Equally, the results depicted connections between cyanidine-3,5-diglucoside and protocatechic acid (r = 0.631; *p* < 0.05), gallic acid derivatives (r = 0.581; *p* < 0.01), chlorogenic acid (r = 0.583; *p* < 0.05), ellagic acid I derivatives (r = 0.660; *p* < 0.05), and cyanidin-3- glucoside (r = 0.972; *p* < 0.01). They showed also ties between cyanidin-3-arabinoside and anthocyanins (r = 0.636; *p* < 0.05), cyanidine-3-glucoside (r = 0.984; *p* < 0.01) as well as cyanidine 3,5 diglucoside (r = 0.956; *p* < 0.01). Relations between the following variables were also manifested by the same study: Cyanidine-3-glucoside and anthocyanins (r = 0.656; *p* < 0.05), rutin and syringic acid (r = 0.705; *p* < 0.05), and finally quercetin-3-glucoside and quercetin-3-galactoside (r = 0.606; *p* < 0.05). Regarding color indices, L* revealed positive links with total phenols (r = 0.713 **) and condensed tannins (r = 0.591; *p* < 0.05), and similarly, b* with a* (r = 0.936; *p* < 0.01). However, a* showed negative links with gallic acid (r = −0.576; *p* < 0.05), protocatechic acid (r = −0.607; *p* < 0.05), and L* (r = −0.727; *p* < 0.01). Unsteadily, c* conveyed negative connections with both protocatechic acid (r = −0.609*), whereas, the hue angle, h°, was negatively linked to total phenols a* (r = −0.646; *p* < 0.05) and c* (r = −0.630; *p* < 0.01), and positive ones with total phenols (r = 0.646; *p* < 0.05) and L* (r = 0.943; *p* < 0.05). In the current study, the results of the anthocyanins were significantly correlated with the ABTS assay. However, no significant correlation was found between the total phenols content and ABTS assay. These results must be interpreted with caution as the Folin–Ciocalteu method used overestimates the concentration of phenolic containing compounds such as ascorbic acids and vitamins could interfere during TP evaluation and that do not give significant correlation. In addition, the synergism between the antioxidants in the mixture makes the antioxidant capacity not only dependent on the concentration, but also on the structure and the interaction between the antioxidants. However, different works have reported good linear correlations between antioxidant activity test and total phenols [12,39,40,41]. The correlation coefficients may provide information on the parameters that are potentially important in assessing strawberry tree genotypes [42]. Significant and strong correlated traits can be used to predict other ones and could be considered of importance for genotypes characterization and discrimination [43].

### 3.9. Principal Components Analysis (PCA)

PCA based on correlation coefficients was used to discriminate between variables in the datasets. The aim of this analysis was to determine the main factors to reduce the number of effective parameters to use in classification of the strawberry tree genotypes based on their biochemical parameters. In our study, only a principal component loading of more than |0.5| was considered as being significant for each factor.

Total variance of 93.19% was explained by seven components (Table 7). The first three components consisted of 26 variables, which explained 68.77% of the total variability observed, which means that these characters had the highest variation between the genotypes and had the highest impact on discrimination of them. The first component accounted for 36.90% of the total variance, which is strongly influenced by the protocatechuic (0.97), gallic acid (0.87), gallocatechin (0.89), gallic acid derivative (0.89), chlorogenic acid (0.83), syringic acid (0.86), ellagic acid derivative I (0.76), ellagic acid derivative II (0.86), ellagic acid (0.72), cyanidin-3-glucoside (0.59), rutin (0.51), cyanidin-3,5-diglucoside (0.70), cyanidin-3-arabinoside (0.57), a* (−0.58), b* (−0.53), and Chroma c* (−0.57). The second component accounted for 18.00% of the total variance and is mainly influenced by total phenols (−0.60), lightness coordinate L* (−0.85), a* (0.65), Chroma c* (0.55), and the hue angle h° (−0.81). The third component represents 13.87% of the total variation, which is defined essentially by total phenols (0.57), condensed tannins (0.77), total anthocyanins (0.80), cyanidin-3-glucoside (0.64), and cyanidin-3-arabinoside (0.64). Generally, these results were in accordance with those reported in previous strawberry tree biochemical studies [30,33]. They have reported that the biochemical attributes are important in order to evaluate the variation in traits of strawberry tree genotypes. These parameters can be used as a useful tool for selecting genotypes for breeding programs or to recommend new cultivars with superior traits.

**Figure 2:**
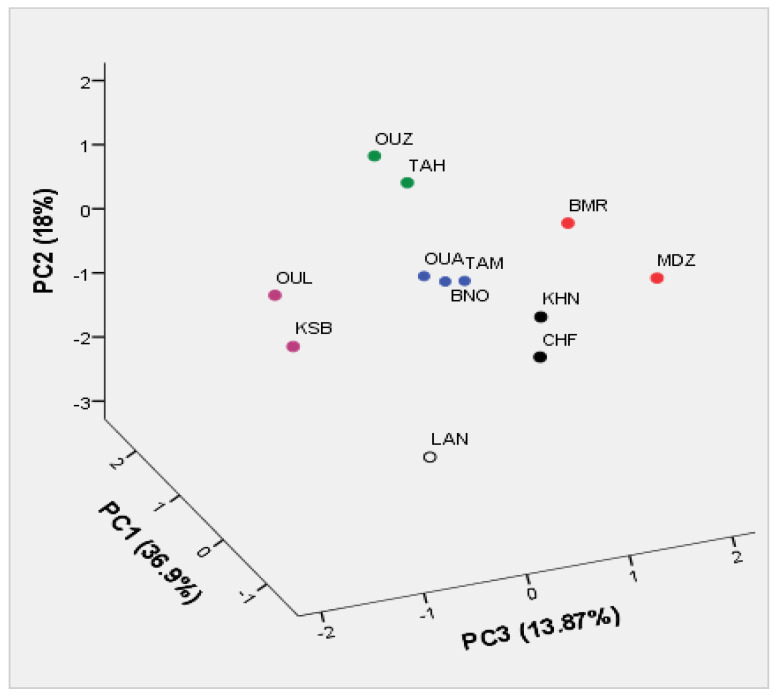
Scatter plot for the first three principal components (PC1/PC2/PC3, 68.77% of total variance) for the studied strawberry tree genotypes based on their biochemical parameters.

Scatter plot was prepared according to the first three principal components: PC1, PC2, and PC3, (36.90, 18, and 13.87% of total variance, respectively) that discriminate between the genotypes according to their chromatic coordinates and biochemical characteristics (Figure 2). Starting from negative to positive values of PC1, the distribution of genotypes indicated a decrease in the peel lightness, total phenols, and condensed tannins. Whereas, starting from negative to positive values of PC2, the most of phenolic compound increased in their values. However, it showed a decrease in the skin coordinates color a*, b*, and c*. Starting from negative to positive values of PC3, the distribution of genotypes indicated an increase in the total anthocyanins, total flavonoids, hydrolyzable tannins, and ABTS.

Generally, these results were in accordance with those reported in previous strawberry tree biochemical studies [30,33]. These studies indicated that high diversity in biochemical traits could be used as an efficient marker system to discriminate between strawberry tree genotypes, comparing our results to other fruits such as sweet cherry [44]. The authors have reported the importance of biochemical characterization as main factor in discriminating and assessing breeding materials of sweet cherry trees. Furthermore, the selection of highly discriminant variables is important to optimize resources for a feasible biochemical assessment. This is especially important in strawberry trees with hundreds of genotypes described worldwide in which many homonymies and synonymies may be detected.

### 3.10. Cluster Analysis

**Figure 3:**
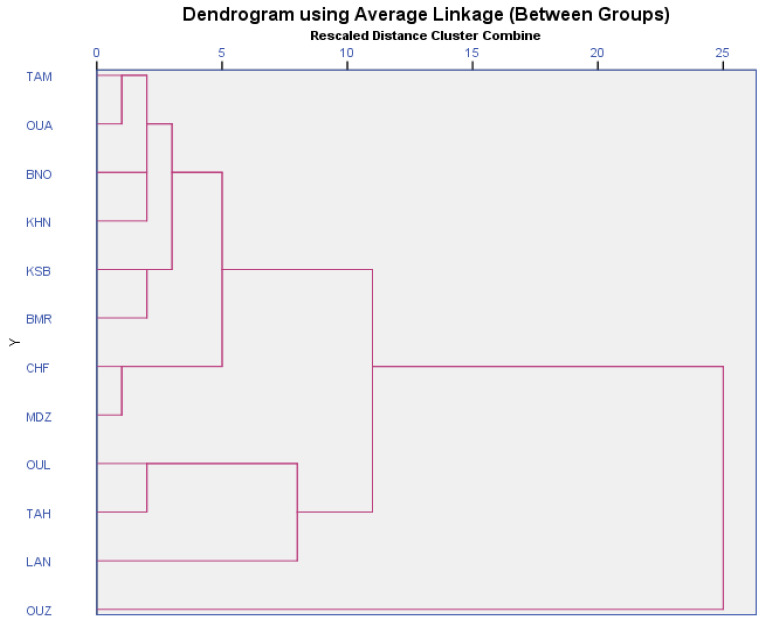
Cluster analysis of the studied genotypes based on the biochemical analysis using squared Euclidian distance method.

Multivariate analysis based on bioactive compounds and antioxidant activity showed high polymorphism among the studied strawberry tree genotypes. Unweighted pair group method (UPGMA) cluster analysis using Euclidean distance coefficient was performed to highlight the similarities among and differences between these genotypes. The genotypes were divided into one main cluster, with a single branch (Figure 3). The genotype “OUZ” was totally discriminated from the cluster. Furthermore, in the main cluster, the genotype “LAN” was the most interesting of the other genotypes and was classified as a singular item. The cluster included 11 genotypes subdivided into four main subgroups. The first subgroup contained “OUL” and “TAH”. The second subgroup was comprised “CHF” and “MDZ”. The third subgroup contained “KSB” and “BMR”. The last subgroup was composed of “TAM”, “OUA”, “BNO”, and “KHN”. The findings of the present study showed the high variability within the strawberry tree genotypes based on biochemical parameters.

## 4. Conclusions

This study proved a high variability among the genotypes studied. The results obtained showed that the strawberry tree fruits are an important source of bioactive compounds. Seventeen phenolic compounds were identified by HPLC, of which gallocatechol and catechin were the most abundant ones. According to the results obtained, the fruits of strawberry tree can be considered as a very rich source of health-promoting compounds, a fact that may encourage many people to consume them as an alternative source of bioactive compounds. The biochemical composition of the fruits of strawberry tree could also be useful to improve their future pharmacological and cosmetic usages. Furthermore, the findings confirmed the usefulness and the importance of biochemical parameters and their complementary information to study diversity within the wild inheritance of strawberry tree. Therefore, the results found in this study may be useful to promote the cultivation of species so as to maintain its longevity and diversity as well as to facilitate its use in breeding programs and industrial valorization. The high variability in biochemical composition observed among genotypes could be attributed to genetic factors. Therefore, it will be important to study and identify the genes responsible for the biochemical properties in order to understand the pattern of variation in the biochemical composition of strawberry tree genotypes.

## Figures and Tables

**Table 1:** Origins geographic of the different samples analysed.

Geographical Origin	Code	Collected Samples (Number)	Zone	Altitude (m)
**Chefchaouen**	CHF	3	Rif	534
**Ouazzane**	OUZ	3	Rif	272
**Moulay Driss Zerhoun**	MDZ	3	Middle Atlas	820
**Laanoucer**	LAN	3	Middle Atlas	1700
**Oulmes**	OUL	3	Middle Atlas	835
**Bab Marzouka**	BMR	3	Rif-Middle Atlas	801
**Khenifra**	KHN	3	Middle Atlas	1390
**El Ksiba**	KSB	3	Middle Atlas	1360
**Bin El-Ouidane**	BNO	3	High Atlas	1420
**Ouaouizerth**	OUA	3	Middle-High Atlas	1050
**Tamscart**	TAM	3	Middle Atlas	1520
**Tahnaout**	TAH	3	High Atlas	1200

**Table 2:** Peak number and wavelength of phenolic compounds.

Phenolic Compounds	Peak Number	Wavelenght (nm)
**Gallic Acid**	1	280
**Protocatechuic**	2	280
**Gallocatechin**	3	280
**Gallic acid Derivative**	4	280
**Catechin**	5	280
**Cholorgenic Acid**	6	280
**Syringic Acid**	7	280
**Ellagic Acid Derivative I**	8	280
**Ellagic Acid Derivative II**	9	280
**Ellagic Acid**	10	280
**Quercetin-3-xyloside**	11	360
**Rutin**	12	360
**Quercetin-3-galactoside**	13	360
**Quercetin-3-glucoside**	14	360
**Cyanidin-3,5-diglucoside**	15	520
**Cyanidin-3-glucoside**	16	520
**Cyanidin-3-arabinoside**	17	520

**Table 3:** Phenolic compound (total phenols, total flavonoids, total anthocyanins, condensed and hydrolyzable tannins) and IC50 value of ABTS of twelve strawberry genotypes.

Genotypes Code	TP (mg GAE/g DW)	TF (mg RE/g DW)	CT (mg TAE/g DW)	HT (mg TAE/g DW)	TA (mg C3,G/100 g DW)	ABTS (mg AAE/g DW)
**TAM**	29.08 ± 7.03 abc	8.26 ± 1.04 d	13.46 ± 1.75 bc	5.65 ± 6.25	0.24 ± 0.15 abc	1.75 ± 0.25 a
**BNO**	31.91 ± 0.89 bcd	7.14 ± 0.74 cd	10.41 ± 1.07 ab	5.41 ± 1.45	0.52 ± 0.23 cd	10.58 ± 2.76 de
**OUA**	22.63 ± 1.74 a	7.68 ± 0.77 cd	12.45 ± 1.70 abc	4.35 ± 1.32	0.12 ± 0.06 a	14.83 ± 3.71 e
**CHF**	28.71 ± 7.34 abc	4.49 ± 0.87 ab	13.54 ± 2.01 bc	6.34 ± 3.47	0.30 ± 0.14 abc	3.33 ± 1.13 ab
**OUZ**	33.97 ± 1.93 cd	4.60 ± 1.06 ab	12.29 ± 1.45 abc	5.51 ± 2.28	0.38 ± 0.15 abcd	2.83 ± 1.46 a
**KSB**	25.37 ± 5.60 ab	3.30 ± 0.60 a	11.62 ± 1.51 a	5.14 ± 3.14	0.15 ± 0.09 ab	4.83 ± 1.88 abc
**OUL**	25.83 ± 2.55 ab	6.96 ± 1.07 cd	11.08 ± 1.63 ab	5.93 ± 2.47	0.16 ± 0.09 ab	8.08 ± 3.64 bcd
**MDZ**	34,72 ± 6.53 cd	6.09 ± 0.88 bc	15.58 ± 1.49 c	6.30 ± 1.06	0.64 ± 0.20 d	19.58 ± 4.49 f
**LAN**	39.06 ± 2.44 d	5.07 ± 1.04 b	16.08 ± 1.50 c	5.88 ± 3.06	0.18 ± 0.09 ab	2.25 ± 0.90 a
**KHN**	32.00 ± 3.67 bcd	8.62 ± 1.10 d	14.66 ± 2.20 bc	5.05 ± 3.68	0.35 ± 0.08 abc	3.08 ± 1.13 ab
**TAH**	27.07 ± 0.96 abc	7.07 ± 0.67 cd	13.09 ± 1.19 abc	4.08 ± 2.43	0.43 ± 0.23 bcd	9.08 ± 3.01 cd
**BMR**	31.80 ± 0.69 bcd	8.04 ± 0.78 d	14.59 ± 1.88 bc	4.77 ± 1.85	0.66 ± 0.15 d	9.58 ± 4.31 cd
**Mean**	30.20	6.44	13.03	5.37	0.34	7.49
**Std. Deviation**	5.70	1.83	2.78	2.60	0.22	5.88
**ANOVA** **Mean Square**	64.00 **	8.83 ***	13.23 *	1.56 NS	0.11 **	93.51 ***

* denotes significant of difference at level 0.05; ** denotes significant of difference at level 0.01; *** denotes significant of difference at level 0.001; NS: Not Significant; data values are means ± SD; values in bold represent, in each column, the minimum and the maximum for each variable; different letters (a–f) in the columns represent statistically significant differences among genotypes according to Duncan’s multi-range test at *p* ˂ 0.05; TP: Total phenols; TF: Total flavonoids; CT: Condensed tannins; HT: Hydrolyzable tannins; TA: Total anthocyanins; GAE: Gallic acid equivalent; RE: Rutin equivalent; TAE: Ttannic acid equivalent; C3,G: Cyanidin-3-glucoside equivalent; AAE: Ascorbic acid equivalent.

**Table 4:** Polyphenolic compounds of twelve strawberry genotypes (mean ± SD in mg GAE/100 g DW).

**Genotypes Code**	**GA**	**PC**	**GC**	**GAD**	**CAT**	**CA**	**SA**	**EADI**	**EADII**	**EA**	**C3G**
TAM	11.75 ± 0.01 e	2.95 ± 0.00 f	43.20 ± 0.08 f	10.56 ± 0.01 h	37.46 ± 0.07 h	17.41 ± 0.00 g	7.68 ± 0.00 e	17.12 ± 0.01 g	17.83 ± 0.01 g	20.86 ± 0.01 h	0.57 ± 0.00 c
BNO	15.37 ± 0.00 g	2.17 ± 0.00 c	27.56 ± 0.02 c	8.57 ± 0.00 d	13.99 ± 0.02 a	18.92 ± 0.01 h	7.41 ± 0.00 d	15.47 ± 0.00 f	13.49 ± 0.01 d	21.09 ± 0.02 i	0.70 ± 0.00 d
OUA	12.52 ± 0.00 f	2.11 ± 0.00 b	40.35 ± 0.01 e	9.61 ± 0.00 f	29.70 ± 0.01 f	14.11 ± 0.00 d	4.87 ± 0.00 b	14.22 ± 0.00 d	13.94 ± 0.00 e	16.91 ± 0.00 e	0.36 ± 0.00 a
CHF	6.09 ± 0.00 b	2.57 ± 0.01 e	16.15 ± 0.03 a	4.98 ± 0.00 a	49.36 ± 0.01 k	5.55 ± 0.00 a	4.27 ± 0.00 a	13.32 ± 0.01 c	8.97 ± 0.01 a	8.42 ± 0.01 a	2.27 ± 0.00 e
OUZ	58.07 ± 0.02 l	6.98 ± 0.01 k	79.88 ± 0.07 l	22.02 ± 0.01 l	65.53 ± 0.04 l	30.25 ± 0.02 l	16.55 ± 0.00 k	30.88 ± 0.04 l	36.56 ± 0.03 k	36.38 ± 0.03 k	6.15 ± 0.00 i
KSB	21.88 ± 0.01 i	3.14 ± 0.01 g	45.23 ± 0.05 g	10.15 ± 0.01 g	33.60 ± 0.03 g	14.50 ± 0.00 e	7.40 ± 0.01 d	18.59 ± 0.01 i	15.96 ± 0.01 f	18.00 ± 0.00 f	0.43 ± 0.01 b
OUL	10.93 ± 0.01 d	4.81 ± 0.00 i	56.81 ± 0.02 i	14.25 ± 0.01 j	19.40 ± 0.01 b	23.73 ± 0.01 j	9.10 ± 0.00 i	19.31 ± 0.01 j	21.65 ± 0.01 j	39.29 ± 0.01 l	0.69 ± 0.00 d
MDZ	4.56 ± 0.02 a	1.84 ± 0.00 a	17.11 ± 0.07 b	7.36 ± 0.01 c	38.98 ± 0.05 j	12.10 ± 0.01 b	6.17 ± 0.01 c	17.22 ± 0.05 h	9.40 ± 0.04 b	14.34 ± 0.02 d	5.68 ± 0.01 h
LAN	35.83 ± 0.02 j	4.18 ± 0.03 h	58.79 ± 0.33 j	7.30 ± 0.01 b	22.09 ± 0.08 c	12.48 ± 0.02 c	7.94 ± 0.02 h	8.05 ± 0.03 b	9.40 ± 0.10 b	10.27 ± 0.05 c	0.57 ± 0.02 c
KHN	7.42 ± 0.00 c	2.32 ± 0.00 d	34.00 ± 0.01 d	9.25 ± 0.00 e	29.47 ± 0.01 e	14.61 ± 0.00 f	7.84 ± 0.00 g	7.79 ± 0.01 a	9.91 ± 0.00 c	10.16 ± 0.01 b	3.04 ± 0.00 f
TAH	36.93 ± 0.02 k	5.90 ± 0.01 j	65.31 ± 0.04 k	14.54 ± 0.02 k	24.68 ± 0.08 d	27.42 ± 0.02 k	7.80 ± 0.01 f	25.06 ± 0.04 k	21.39 ± 0.02 i	33.73 ± 0.02 j	7.21 ± 0.01 j
BMR	15.45 ± 0.00 h	4.18 ± 0.00 h	54.35 ± 0.02 h	12.75 ± 0.00 i	38.55 ± 0.01 i	20.74 ± 0.00 i	11.85 ± 0.00 j	14.95 ± 0.01 e	19.01 ± 0.01 h	19.50 ± 0.01 g	4.24 ± 0.00 g
Mean	19.73	3.6	44.9	10.95	33.57	17.65	8.24	16.83	16.46	20.75	2.66
Std. deviation	15.57	1.59	18.76	4.37	13.63	6.79	3.16	6.26	7.62	10.12	2.48
ANOVA mean square	771.20 ***	8.06 ***	1119.71 ***	60.68 ***	591.06 ***	146.73 ***	31.75 ***	124.88 ***	184.85 ***	325.95 ***	19.49 ***
**Genotypes Code**	**Q3X**		**RT**		**Q3GA**		**Q3G**		**C3,5DG**		**C3A**
TAM	3.96 ± 0.01 h		1.42 ± 0.01 i		3.40 ± 0.02 i		3.21 ± 0.01 g		n.d		n.d
BNO	3.24 ± 0.01 g		0.75 ± 0.00 b		1.33 ± 0.01 b		2.66 ± 0.01 def		n.d		n.d
OUA	2.08 ± 0.00 c		0.67 ± 0.00 a		1.42 ± 0.00 c		1.54 ± 0.00 b		n.d		n.d
CHF	2.11 ± 0.01 cd		1.17 ± 0.00 g		1.66 ± 0.00 d		2.11 ± 0.01 cd		0.61 ± 0.00 a		0.36 ± 0.01 a
OUZ	7.92 ± 0.04 k		1.70 ± 0.01 j		2.82 ± 0.01 g		2.90 ± 0.01 efg		2.62 ± 0.01 e		1.31 ± 0.02 e
KSB	4.09 ± 0.01 i		1.06 ± 0.01 e		3.46 ± 0.02 j		2.89 ± 0.00 efg		n.d		n.d
OUL	2.14 ± 0.00 d		1.43 ± 0.00 i		1.79 ± 0.01 e		1.71 ± 0.01 bc		n.d		n.d
MDZ	1.43 ± 0.01 a		0.96 ± 0.00 d		3.02 ± 0.01 h		2.12 ± 0.01 cd		1.59 ± 0.02 c		1.07 ± 0.00 d
LAN	2.72 ± 0.03 e		1.26 ± 0.01 h		3.03 ± 0.04 h		2.54 ± 0.02 de		n.d		n.d
KHN	1.64 ± 0.00 b		1.10 ± 0.00 f		1.00 ± 0.01 a		0.98 ± 0.00 a		0.67 ± 0.00 b		0.89 ± 0.00 b
TAH	2.81 ± 0.03 f		0.90 ± 0.02 c		2.73 ± 0.02 f		2.27 ± 0.01 d		3.30 ± 0.02 f		1.64 ± 0.01 f
BMR	6.31 ± 0.01 j		2.26 ± 0.01 k		1.35 ± 0.00 b		3.10 ± 1.02 fg		1.63 ± 0.00 d		1.01 ± 0.01 c
Mean	3.37		1.22		2.25		2.34		0.87		0.52
Std.deviation	1.91		0.43		0.88		0.7		1.12		0.6
ANOVA mean square	11.58 ***		0.59 ***		2.46 ***		1.38 ***		4.02 ***		1.14 ***

*** denotes significant of difference at level 0.001; data values are means ± SD; values in bold represent, in each column, the minimum and the maximum for each variable; n.d: Not determined; different letters (a–l) in columns represent statistically significant differences among genotypes according to Duncan’s multi-range test at *p* < 0.05 GA: Gallic acid; PC: Protocatechuic; GC: Gallocatechin; GAD: Gallic acid derivative; CAT: Catechin; CA: Cholorgenic acid; SA: Syringic acid; EADI: Ellagic acid derivative I; EADII: Ellagic acid derivative II; EA: Ellagic acid; C3G: Cyanidin-3-glucoside; Q3X: Quercetin-3-xyloside; RT: Rutin; Q3GA: Quercetin-3-galactoside; Q3G: Quercetin-3-glucoside; C3,5D: Cyanidin-3,5-diglucoside; C3A: Cyanidin-3-arabinoside; GAE: Gallic acid equivalent.

**Table 5:** Colorimetric characters of twelve strawberry genotypes.

Genotypes Code	L*	a*	b*	c*	h°
**TAM**	30.30 ± 3.99 a	58.91 ± 15.96 a	93.73 ± 20.41 a	110.17 ± 25.69 a	58.71 ± 1.53 a
**BNO**	25.83 ± 9.86 a	51.80 ± 11.55 a	84.51 ± 11.47 a	100.59 ± 15.92 a	54.80 ± 7.29 a
**OUA**	26.27 ± 9.44 a	54.87 ± 12.57 a	89.69 ± 16.76 a	106.18 ± 21.82 a	54.70 ± 6.42 a
**CHF**	35.47 ± 15.78 a	48.18 ± 19.71 a	86.14 ± 13.37 a	100.63 ± 21.13 a	58.09 ± 11.90 a
**OUZ**	37.79 ± 15.29 a	39.47 ± 21.25 a	77.88 ± 14.14 a	89.36 ± 21.95 a	60.78 ± 14.24 a
**KSB**	33.32 ± 10.60 a	40.79 ± 17.11 a	73.88 ± 16.05 a	86.08 ± 22.60 a	58.36 ± 10.06 a
**OUL**	32.18 ± 3.16 a	38.39 ± 13.38 a	70.85 ± 12.82 a	82.20 ± 17.91 a	58.14 ± 9.17 a
**MDZ**	35.03 ± 16.17 a	48.38 ± 16.54 a	84.92 ± 6.54 a	99.62 ± 13.08 a	57.29 ± 11.74 a
**LAN**	50.78 ± 3.44 a	28.93 ± 15.10 a	70.84 ± 7.65 a	78.30 ± 12.00 a	66.45 ± 12.38 a
**KHN**	33.42 ± 21.21 a	44.37 ± 21.28 a	81.68 ± 12.34 a	95.05 ± 20.63 a	58.81 ± 13.83 a
**TAH**	32.65 ± 5.19 a	38.19 ± 11.84 a	74.16 ± 10.22 a	85.08 ± 14.64 a	59.29 ± 8.82 a
**BMR**	39.09 ± 5.01 a	46.38 ± 17.55 a	86.23 ± 14.16 a	98.21 ± 19.41 a	59.91 ± 8.95 a
**Mean**	34.34	44.89	81.21	94.29	58.78
**Std. Deviation**	11.43	15.85	13.39	18.71	9.00
**ANOVA** **Mean Square**	128.11 NS	206.61 NS	172.51 NS	298.96 NS	27.30 NS

NS: Not significant; data values are means ± SD; values in bold represent, in each column, the minimum and the maximum for each variable. (L*: Lightness, a*: Color variation from red to green, b*: Color variation from yellow to blue, c*: Chroma, h°: Hue).

**Table 6:** Correlation coefficients among biochemical parameters analyzed.

	TP	TF	HT	CT	ANT	ABTS	GA	PC	GC	GAD	CAT	CA	SA	EADI	EADII	EA	C3G	RT	Q3GA	Q3G	C3,5	C3A	L*	a*	b*	c*	h*
**TP**	1																										
**TF**	−0.121	1																									
**HT**	0.451	−0.395	1																								
**CT**	0.631 *	0.278	0.193	1																							
**ANT**	0.438	0.249	0.000	0.337	1																						
**ABTS**	−0.163	0.226	−0.137	0.053	0.444	1																					
**GA**	0.295	−0.401	−0.235	−0.080	−0.067	−0.354	1																				
**PC**	0.113	−0.219	−0.209	−0.078	−0.017	−0.347	0.841 **	1																			
**GC**	0.058	−0.080	−0.394	−0.108	−0.186	−0.356	0.834 **	0.913 **	1																		
**GAD**	−0.058	−0.011	−0.321	−0.267	0.058	−0.162	0.717 **	0.854 **	0.841 **	1																	
**CAT**	0.142	−0.382	0.226	0.143	0.150	−0.225	0.334	0.306	0.150	0.388	1																
**CA**	−0.052	0.174	−0.426	−0.283	0.175	−0.050	0.651 *	0.812 **	0.806 **	0.927 **	0.077	1															
**SA**	0.343	−0.073	−0.090	−0.052	0.250	−0.292	0.705 *	0.771 **	0.764 **	0.870 **	0.465	0.770 **	1														
**EADI**	−0.198	−0.268	−0.167	−0.414	0.134	0.052	0.619 *	0.710 **	0.564	0.821 **	0.456	0.769 **	0.590 *	1													
**EADII**	−0.096	−0.107	−0.235	−0.347	0.022	−0.207	0.718 **	0.839 **	0.800 **	0.976 **	0.464	0.883 **	0.849 **	0.872 **	1												
**EA**	−0.272	0.045	−0.228	−0.451	−0.018	0.018	0.495	0.757 **	0.692 *	0.849 **	0.036	0.906 **	0.590 *	0.822 **	0.847 **	1											
**C3G**	0.259	0.006	−0.199	0.342	0.656 *	0.227	0.402	0.496	0.272	0.476	0.453	0.469	0.429	0.553	0.409	0.316	1										
**RT**	0.289	0.051	0.105	0.228	0.273	−0.344	0.226	0.487	0.465	0.457	0.447	0.339	0.705 *	0.172	0.466	0.228	0.216	1									
**Q3GA**	0.179	−0.501	0.201	0.000	−0.157	−0.168	0.413	0.286	0.294	0.162	0.244	0.105	0.140	0.393	0.222	0.142	0.120	0.051	1								
**Q3G**	0.227	−0.314	0.090	−0.169	0.203	−0.237	0.406	0.308	0.308	0.252	0.287	0.251	0.411	0.393	0.382	0.177	0.032	0.453	0.606 *	1							
**C3,5**	0.163	−0.036	−0.316	0.226	0.538	0.139	0.546	0.631 *	0.424	0.581 *	0.435	0.583 *	0.475	0.660 *	0.529	0.435	0.972 **	0.202	0.171	0.107	1						
**C3A**	0.238	0.093	−0.298	0.358	0.636 *	0.162	0.382	0.484	0.295	0.479	0.421	0.473	0.442	0.478	0.389	0.277	0.984 **	0.243	0.032	−0.038	0.956 **	1					
L*	0.713 **	−0.389	0.310	0.591 *	0.019	−0.369	0.414	0.370	0.348	−0.002	0.197	−0.098	0.306	−0.193	−0.037	−0.220	0.158	0.472	0.332	0.213	0.138	0.154	1				
a*	−0.379	0.470	−0.049	−0.115	0.158	0.301	−0.576 *	−0.607 *	−0.560	−0.262	0.095	−0.252	−0.341	−0.092	−0.181	−0.218	−0.208	−0.176	−0.232	0.046	−0.258	−0.214	−0.727 **	1			
b*	−0.145	0.471	−0.012	0.182	0.265	0.226	−0.498	−0.566	−0.527	−0.296	0.251	−0.329	−0.265	−0.195	−0.223	−0.382	−0.094	−0.011	−0.233	0.091	−0.163	−0.090	−0.469	0.936 **	1		
c*	−0.224	0.459	−0.009	0.078	0.233	0.270	−0.539	−0.609 *	−0.576	−0.305	0.205	−0.328	−0.314	−0.168	−0.229	−0.347	−0.130	−0.107	−0.254	0.043	−0.198	−0.131	−0.578 *	0.972 **	0.990 **	1	
h*	0.646 *	−0.301	0.189	0.506	−0.124	−0.554	0.541	0.507	0.528	0.138	0.130	0.075	0.379	−0.102	0.100	−0.065	0.123	0.459	0.424	0.264	0.152	0.136	0.943 **	−0.747 **	−0.524	−0.630 *	1

*. Correlation is significant at the 0.05 level; **. Correlation is significant at the 0.01 level; TP: Total phenols; TF: Total flavonoids; HT: Hydrolyzable tannins; CT: Condensed tannins; TA: Total anthocyanins; GA: Gallic acid; PC: Protocatechuic; GC: Gallocatechin; GAD: Gallic acid derivative; CAT: Catechin; CA: Cholorgenic acid; SA: Syringic acid; EADI: Ellagic acid derivative I; EADII: Ellagic acid derivative II; EA: Ellagic acid; C3G: Cyanidin-3-glucoside; RT: Rutin; Q3GA: Quercetin-3-galactoside; Q3G: Quercetin-3-glucoside; C3,5: Cyanidin-3,5-diglucoside; C3A: Cyanidin-3-arabinoside.

**Table 7:** Eigenvectors of principal component axes from PCA analysis of studied variables.

Variables	Component						
	1	2	3	4	5	6	7
**Total Phenols**	0.219	**−0.597**	**0.575**	0.051	0.085	0.299	-0.060
**Total Flavonoids**	−0.257	0.470	0.237	−0.287	**0.679**	0.108	0.160
**Hydrolyzable Tannins**	−0.195	−0.482	0.142	0.413	−0.240	0.199	**−0.547**
**Condensed Tannins**	−0.063	−0.411	**0.770**	−0.168	0.219	−0.123	0.153
**Total Anthocyanins**	0.122	0.273	**0.796**	−0.090	−0.060	0.427	−0.103
**ABTS**	−0.278	0.453	0.260	−0.353	−0.336	0.385	−0.011
**Gallic Acid**	**0.871**	−0.147	−0.107	0.040	−0.086	−0.092	0.220
**Protocatechuic**	**0.966**	−0.024	−0.115	−0.055	0.075	−0.068	−0.009
**Gallocatechin**	**0.888**	−0.040	−0.263	−0.054	0.282	−0.043	0.191
**Gallic Acid Derivative**	**0.888**	0.365	−0.124	0.059	0.146	−0.048	−0.098
**Catechin**	0.394	0.074	0.399	**0.578**	−0.210	−0.488	−0.208
**Chlorogenic Acid**	**0.829**	0.430	−0.142	−0.129	0.193	0.195	0.020
**Syringic Acid**	**0.858**	0.070	0.110	0.214	0.293	0.094	−0.200
**Ellagic Acid Derivative I**	**0.757**	0.483	−0.110	0.182	−0.363	0.032	−0.033
**Ellagic Acid Derivative II**	**0.864**	0.377	−0.168	0.231	0.085	−0.040	−0.103
**Ellagic Acid**	**0.719**	0.450	−0.355	−0.084	0.024	0.207	−0.152
**Cyanidin-3-Glucoside**	**0.590**	0.255	**0.642**	−0.265	−0.284	−0.104	0.001
**Rutin**	**0.509**	−0.130	0.291	0.382	**0.509**	0.073	−0.218
**Quercetin-3-Galactoside**	0.368	−0.299	−0.079	0.392	−0.465	0.159	0.461
**Quercetin-3-Glucoside**	0.382	−0.061	0.033	**0.692**	−0.065	0.416	0.345
**Cyanidin-3,5-Diglucoside**	**0.696**	0.285	0.494	−0.254	−0.274	−0.145	0.102
**Cyanidin-3-Arabinoside**	**0.575**	0.258	**0.640**	−0.323	−0.168	−0.178	0.021
L*	0.379	**−0.854**	0.287	−0.004	0.088	−0.012	0.034
a*	**−0.579**	**0.650**	0.165	0.430	0.091	−0.015	0.106
b*	**−0.529**	0.485	0.419	0.470	0.186	−0.114	0.160
c*	**−0.572**	**0.550**	0.341	0.447	0.124	−0.097	0.119
h*	0.493	**−0.811**	0.133	0.004	0.195	−0.048	0.180
**% of Variance**	360.90	180.00	130.87	90.40	60.96	40.17	30.89
**Cumulative %**	360.90	540.90	680.77	780.18	850.14	890.31	930.20

Eigenvalues higher than |0.5| are marked in bold.
